# Aberrant right subclavian artery- suggested mechanism for esophageal foreign body impaction: Case report

**DOI:** 10.1186/1749-7922-6-12

**Published:** 2011-04-09

**Authors:** Eran Brauner, Moshe Lapidot, Ran Kremer, Lael A Best, Yoram Kluger

**Affiliations:** 1 Department of general surgery, Rambam Health Care Campus, Haifa, Israel; 2 Department of Thoracic surgery, Rambam Health Care Campus, Haifa, Israel

## Abstract

Aberrant right subclavian artery (ARSA) is asymptomatic in most cases. This variant anatomy can cause dysphagia in elderly patients. Impaction of foreign body in the esophagus is rarely the presenting symptom of ARSA. We present an eighty four years old patient who first presented with esophageal foreign body impaction and was diagnosed with an aberrant right subclavian artery compressing the esophagus just below the site of impaction.

We assume that the exact place of impaction was not incidental and that a relative narrowing of the esophagus caused by the vascular anomaly is responsible for this specific presentation.

## Background

In 1794, David Byaford, a young surgeon accidentally discovered an anomalous origin of right subclavian artery in a post mortem study of a 62 years old patient who suffered prolonged dysphagia. He then coined the term "*lusus naturae*" which means "*a freak of nature*". The term "*dysphagia lusoria*" is used to describe dysphagia which originates from extrinsic compression of the esophagus from any vascular anomaly of the aortic arch mostly from aberrant right subclavian artery [1].

Aberrant right subclavian artery represents the most common congenital vascular anomaly of the aortic arch. Its incidence is between 0.5% and 1.8% [2]. The presence of this anomaly is often asymptomatic, and may be discovered incidentally on imaging or at postmortem studies. As many as 60% to 80% of patients remain lifelong symptom-free.

Retention of ingested foreign objects in the esophagus above the level of a vascular anomaly was first described in a series of 4 children, two with vascular ring and two with ARSA who presented with esophageal foreign bodies [3].

We present a case of an elderly patient with aberrant right subclavian artery diagnosed when the patient presented with esophageal foreign body impacted above the vascular anomaly. We suggest a causative relationship between the two.

## Case presentation

An eighty four years old patient was transferred to our emergency department complaining of recent onset dysphagia and odinophagia after accidentally swallowing her prosthetic teeth.

Both firm and flexible esophagoscopy done in the referring institute failed in retrieving the foreign body out. Her past medical history indicated neither chronic dysphagia nor respiratory complains.

The patient suffered from diabetes mellitus hypertension and was on warfarine treatment for paroxysmal atrial fibrillation. She had two episodes of cerebrovascular accident (CVA); the last was 2 months prior to her admission. Residual of left hemiparesis and dysartria were noted.

Upon admission she was alert and hemodynamically stable. Her temperature was 38°C. Physical examination was remarkable for tachypnea and mild desaturation.

Her laboratory results revealed mild leukocytosis.

**Figure 1:**
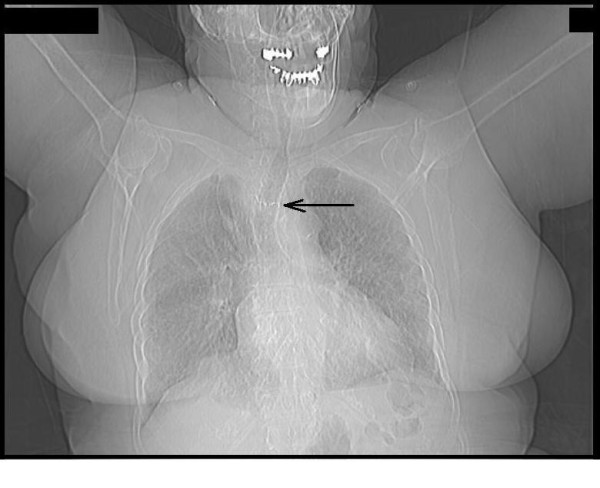
**Chest X ray; arrow pointing at the foreign body in the mid esophagus**.

On plain film a foreign body was seen situated 20 cm from the teeth (Figure 1).

**Figure 2:**
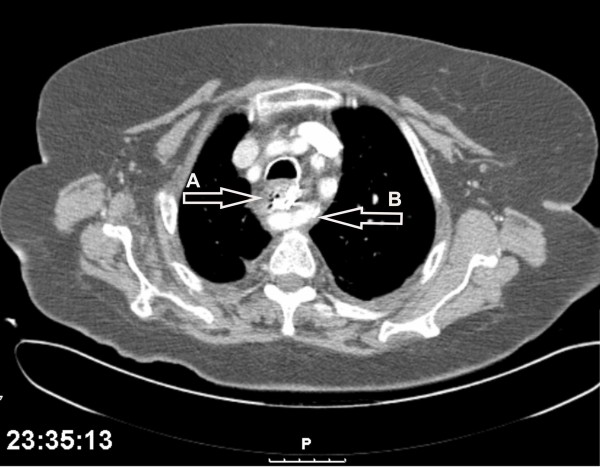
**Tomography of the chest; foreign body (A) situated at the level of the aberrant right subclavian artery (B)**.

A computed tomography (CT) of the neck and chest with swallowed of contrast material revealed (Figure 2) a foreign body composed of metal wire at the level of D3-4. No contrast leak was noted. An aberrant right subclavian artery was seen passing between the esophagus and the vertebra just below the level the foreign body (Figure 2).

Though no contrast leak was noted, the suspicion for esophageal perforation was high and a decision was made for exploration.

On surgical exploration of the neck through a left longitudinal incision, edema and inflammation of the lower neck and the upper mediastinum was encountered suggesting esophageal perforation.

**Figure 3:**
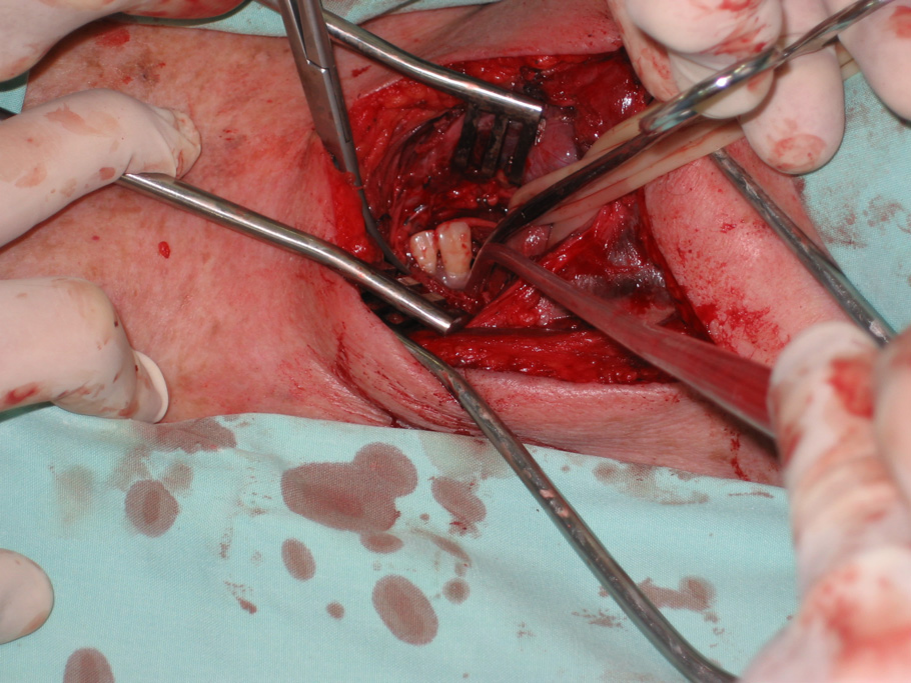
Foreign body revealed at esophagotomy (left side of the picture pointing the feet of the patient).

Tow metal hooks were seen on both sides of the esophagus. Esophagotomy was done and a complex of two prosthetic teeth with two metal hooks extending from its sides piercing the walls of the esophagus was exposed (Figure 3).

The foreign body was extracted carefully. There was no gross spillage of content from the site of the incision. The patient was stable and local conditions allowed the esophagotomy to be closed primarily. A close suction drain was placed after a thorough irrigation.

The patient was transferred to the intensive care unit for further treatment and stabilization. The post operative curse was complicated with a lobar pneumonia from which she never recovered. The patient expired on post operative day 14.

## Discussion

In normal embryologic development, the subclavian arteries originate from the seventh intersegment arteries. The distal segment of the right dorsal aorta degenerates, and the right seventh intersegment artery becomes confluent with the right fourth arch.

In the anomaly of aberrant right subclavian artery, abnormal development results from degeneration of the entire right fourth arch. The right seventh intersegment artery persists in its attachment to the distal descending aorta [4]. In 80% of cases, it crosses between the esophagus and the vertebral column, in 15% of cases it runs between the esophagus and the trachea, and in 5% of cases it passes anterior to both the trachea and esophagus [5].

Aberrant right subclavian artery in the adult patient, usually present with dysphagia. Symptoms are primarily for solid food and are associated with regurgitation, postprandial bloating or chest pain [5]. We could not find reports of ARSA resulting in esophageal foreign body impaction in adults.

The esophagus has 3 areas of narrowing where foreign bodies are most likely to become entrapped: the upper esophageal sphincter (UES), which consists of the cricopharyngeus muscle; the crossover of the aorta; and the lower esophageal sphincter (LES). Ingestion of foreign bodies are much more common in children than in adult and considering the fact that most of the patient harboring an aberrant right subclavian artery are asymptomatic through their life time [5], the association between these two entities could be incidental.

In adults the incidence of foreign body ingestion is rare. It is reasonable to assume that the foreign body in our case was trusted into the esophagus at this exact level because of a relative narrowing caused by the back compression of the right aberrant subclavian artery on the esophagus. Supporting this assumption is the CT scan findings of our patient revealing the foreign body impacted just at the level of the vascular anomaly.

## Conclusion

An aberrant right subclavian artery should be suggested when foreign body in the proximal esophagus is encountered even in the previously asymptomatic patient.

## Abbreviations

ARSA: Aberrant Right Subclavian Artery; CT: Computed Tomography; CVA: Cerebro-Vascular Accident; UES: Upper esophageal sphincter; LES: Lower esophageal sphincter.

## Declaration of competing interests

The authors declare that they have no competing interests.

## Patient consent

Written Informed consent was obtained by the patient's daughter for publication of this case report and any accompanying images. A copy of the written consent is available for review by the editor in chief of this journal.

## Authors' contributions

EB - conceived the study and participated in its design, ML - operating surgeon, RK - operating surgeon, LAB - critical review study concept and design, YK - critical review study concept and design. All authors read and approved the final manuscript.

## References

[B1] Asherson N David Bayford, His syndrome and sign of dysphagia lusoria Annals of the Royal College of Surgeons of England 1979 61 63 67 369446 PMC2494476

[B2] Carrizo GJ Marjani MA Dysphagia lusoria caused by an aberrant right subclavian artery Tex Heart Inst J 2004 31 168 71 15212130 PMC427379

[B3] Currarino G Nikadiho H Esophageal foreign bodies in children with vascular ring or aberrant right subclavian artery: coincidence or causation? Pediatr Radiol 1991 21 406 408 10.1007/BF02026672 1749671

[B4] Bisognano JD Young B Brown JM Gill EA Fang FC Zisman LS Diverse presentation of aberrant origin of the right subclavian artery Chest 1997 112 1693 1697 10.1378/chest.112.6.1693 9404777

[B5] Levitt B Richter JE Dysphagia lusoria: a comprehensive review Diseases of the Esophagus 2007 20 455 460 10.1111/j.1442-2050.2007.00787.x 17958718

